# Memory Concerns, Memory Performance and Risk of Dementia in Patients with Mild Cognitive Impairment

**DOI:** 10.1371/journal.pone.0100812

**Published:** 2014-07-14

**Authors:** Steffen Wolfsgruber, Michael Wagner, Klaus Schmidtke, Lutz Frölich, Alexander Kurz, Stefanie Schulz, Harald Hampel, Isabella Heuser, Oliver Peters, Friedel M. Reischies, Holger Jahn, Christian Luckhaus, Michael Hüll, Hermann-Josef Gertz, Johannes Schröder, Johannes Pantel, Otto Rienhoff, Eckart Rüther, Fritz Henn, Jens Wiltfang, Wolfgang Maier, Johannes Kornhuber, Frank Jessen

**Affiliations:** 1 Department of Psychiatry, University of Bonn, Bonn, Germany; 2 German Center for Neurodegenerative Diseases, Bonn, Germany; 3 Center for Geriatric Medicine, Ortenau Klinikum, Offenburg-Gengenbach, Germany; 4 Department of Gerontopsychiatry, Central Institute of Mental Health, Mannheim, Germany; 5 Department of Psychiatry, Technical University of Munich, Munich, Germany; 6 Department of Neurology, University of Aachen, Aachen, Germany; 7 Department of Psychiatry, University of Göttingen, Göttingen, Germany; 8 Department of Psychiatry, Ludwig Maximilian University, Munich, Germany; 9 Department of Psychiatry, Charité Berlin, Campus Benjamin Franklin, Berlin, Germany; 10 Department of Psychiatry, University of Hamburg, Hamburg, Germany; 11 Department of Psychiatry and Psychotherapy, Medical Faculty, Heinrich-Heine-University, Duesseldorf, Germany; 12 Center for Geriatric Medicine and Gerontology, University of Freiburg, Freiburg, Germany; 13 Department of Psychiatry, University of Leipzig, Leipzig, Germany; 14 Department of Psychiatry, University of Heidelberg, Heidelberg, Germany; 15 Institute of General Practice, University of Frankfurt, Frankfurt am Main, Germany; 16 Department of Medical Informatics, University of Göttingen, Göttingen, Germany; 17 Brookhaven National Laboratory, Upton, New York, United States of America; 18 Department of Psychiatry University of Essen, Essen, Germany; 19 Department of Psychiatry, Friedrich-Alexander-University Erlangen, Erlangen, Germany; University of Barcelona, Spain

## Abstract

**Background:**

Concerns about worsening memory (“memory concerns”; MC) and impairment in memory performance are both predictors of Alzheimer's dementia (AD). The relationship of both in dementia prediction at the pre-dementia disease stage, however, is not well explored. Refined understanding of the contribution of both MC and memory performance in dementia prediction is crucial for defining at-risk populations. We examined the risk of incident AD by MC and memory performance in patients with mild cognitive impairment (MCI).

**Methods:**

We analyzed data of 417 MCI patients from a longitudinal multicenter observational study. Patients were classified based on presence (n = 305) vs. absence (n = 112) of MC. Risk of incident AD was estimated with Cox Proportional-Hazards regression models.

**Results:**

Risk of incident AD was increased by MC (HR = 2.55, 95%CI: 1.33–4.89), lower memory performance (HR = 0.63, 95%CI: 0.56–0.71) and ApoE4-genotype (HR = 1.89, 95%CI: 1.18–3.02). An interaction effect between MC and memory performance was observed. The predictive power of MC was greatest for patients with very mild memory impairment and decreased with increasing memory impairment.

**Conclusions:**

Our data suggest that the power of MC as a predictor of future dementia at the MCI stage varies with the patients' level of cognitive impairment. While MC are predictive at early stage MCI, their predictive value at more advanced stages of MCI is reduced. This suggests that loss of insight related to AD may occur at the late stage of MCI.

## Introduction

The syndrome of mild cognitive impairment [1] (MCI) has been established as a risk state for Alzheimer's Dementia (AD). Patients with MCI show cognitive impairment objectified by neuropsychological testing while their functional activities are largely intact. In addition, current criteria for MCI [1]–[3] require report on cognitive decline, provided either by the patient and/or by an informant or clinician who knows the patient well.

Compared to the current knowledge and standards of neuropsychological testing, the criterion of subjective report about cognitive decline in the definition of MCI is less elaborated. It is unknown whether more precise operationalization (either quantitatively or qualitatively) of this criterion may increase the predictive accuracy for AD in MCI patients. In fact, in everyday clinical practice, the criterion of experienced or observed cognitive decline might often be considered fulfilled by the fact that a patient consults the medical system for diagnostic workup of cognitive impairment. Studies that investigated the role of individual and informant reports for the prediction of AD in MCI are rare. One early study [4] found informant reports but not the individual's memory complaints associated with future AD in memory impaired patients. A recent study [5] in a non-demented elderly community sample found both self and informant reports to be predictive, while in a combined predictive model only informant reports together with neuropsychological tests remained a significant predictor.

Other studies, based on pre-MCI samples, showed elevated risk of future AD [6]–[8] as well as associations with biomarkers of Alzheimer's disease in individuals who report self-experienced cognitive decline [9]–[15]. However, there are also studies that did not find associations of self-reported cognitive decline with either incident AD [16] or biomarkers of Alzheimer's disease [17], [18] in pre-MCI samples. Importantly, comparability of results across studies is limited due to heterogeneity of samples and assessment of self-experienced cognitive decline. Further, it was recently reported that, in individuals with normal cognitive test performance (non-MCI), those who are particularly concerned about their experienced memory decline have a higher risk of developing AD, as compared to those who report a self-experienced memory decline without concerns [19], [20]. Thus, the appraisal of the experienced decline as worrying may be of specific predictive value when assessing an individual's report.

Based on the existing data, the significance of self-reported concerns about worsening memory (hereafter: “memory concerns” (MC)) in MCI is yet unclear and it is largely unknown what factors might influence the report or denial of MC in MCI patients [21], [22]. Reduced self-awareness is one factor that might influence the report of MC in this patient group [22]. Self-awareness often becomes impaired during the progression of Alzheimer's disease. Hence, unawareness (also termed anosognosia) concerning the memory impairment is frequently observed in AD [23]. Reduced self-awareness and anosognosia are also observed in MCI patients [23]–[25]. However, levels of awareness are heterogeneous among these patients [22]. This might contribute to the fact that MC are not consistently present in patients with MCI [23], [24], [26].

The heterogeneity in self-awareness may originate from the fact that anosognosia as a core symptom of AD manifests at the stage of MCI and that the likelihood of its occurrence rises with increasing cognitive impairment. Evidence for this assumption comes from studies that investigated self-awareness in patients with AD and patients with amnestic MCI (i.e. with clinical impairment in the memory domain, evidenced by neuropsychological testing [1], [2]). Patients with advanced amnestic MCI, scoring lower than two standard deviations (SD) below age-corrected norms on a memory test [24], showed symptoms of anosognosia similarly severe compared to the AD group. In a study on amnestic MCI patients, Nobili and colleagues found that low awareness of memory deficits was associated with more progressed Alzheimer's disease pathology [27]. Moreover, results from a recent study showed that cognitive complaints decreased with decreasing cognitive performance in MCI patients, while the relationship was opposite (i.e. reported complaints increased with decreasing memory performance) in individuals with only subjective memory impairment but no MCI [18]. These results suggest that, within the stage of MCI, those patients with more severe cognitive impairment tend to have reduced insight into their cognitive deficits.

Based on the empirical evidence a hypothetical model of AD prediction in MCI can be formulated: At the earliest stage of impairment (early MCI) self-awareness of the patient is mostly unaffected. Here, MC should reflect the true self-perceived, longitudinal intra-individual decline and should contribute to AD prediction in addition to cross-sectional impairment on tests. At later stages of MCI, self-awareness is waning and the predictive value of MC is declining. MC as defined in this model comprises two important aspects, i.e. the specific notion of (1) a decline in memory performance and (2) the appraisal of this self-perceived decline as worrying. The appraisal as worrying extends beyond the subjective report about cognitive decline as part of the general MCI criteria and has been found to be of higher predictive value than the notion of a worsening memory without worries [19], [20]. This clearly separates the definition of memory concerns in our study from subjective memory decline in general.

In the present study, we tested the proposed model in a sample of MCI patients whose memory impairment ranged from very mild to advanced severity.

## Methods

### Ethics statement

The protocol of the study was approved by the Institutional Review Board (IRB) of the Medical Faculty, University of Erlangen (coordinating study center) and by IRBs at each individual participating study center, listed in the following: IRB Medical Faculty, University of Hamburg; IRB Charité – University Medicine Berlin; IRB Medical Faculty, University of Göttingen; IRB Medical Faculty, University of Düsseldorf; IRB Medical Faculty, University of Bonn; IRB Medical Faculty, University of Leipzig; IRB Medical Faculty, University of Frankfurt (am Main); IRB Medical Faculty, University of Heidelberg; IRB Medical Faculty, Saarland University; IRB Medical Faculty, University of Mannheim; IRB Medical Faculty, University of Freiburg; IRB Medical Faculty, Ludwig Maximilian University Munich; IRB Medical Faculty, Technical University Munich.

The study was conducted in accordance with the Declaration of Helsinki. After complete description of the study to the patients, written informed consent was obtained.

### Participants

Subjects were recruited between 2003 and 2007 at 14 specialized university memory clinics collaborating within the German Dementia Competence Network (DCN). The general procedures for assessment and selection of subjects have been reported in detail previously [28]. Briefly, patients over 50 years of age who were referred to or sought help at one of the participating memory clinics underwent a clinical, neuropsychological and laboratory assessment and brain imaging. Patients with either MCI or mild dementia were asked to participate in this longitudinal observational study.

### Clinical and neuropsychological assessment

Patients were assessed annually by experienced physicians and neuropsychologists for up to three years with standardized diagnostic procedures as described in detail previously [28]. This assessment included the neuropsychological test battery of the Consortium to Establish a Registry for Alzheimer's Disease (CERAD-NP) [29]. The CERAD-NP consists of various subtests, including the Mini Mental State Examination (MMSE) [30], and is specifically designed to assess the cognitive domains most commonly affected in AD. The subtests are (in order of administration) (1) Verbal Fluency, (2) modified Boston Naming Test (15 item version), (3) the MMSE, (4) Word List Learning of a 10-item word list (sum of three learning trials; maximum score of 30), (5) Figure Copying (maximum score of 11), (6) Word List Delayed Recall (maximum score of 10), (7) Word List Recognition (maximum score of 10 or 100%), and (8) Figure Recall (maximum score of 11). We used the Word List Delayed Recall subtest (CERAD-DR) as a measure of objective memory impairment as delayed recall of word lists is considered among the tests that are most sensitive to incipient AD [3]. In addition, high levels of diagnostic accuracy for the CERAD-DR have been reported regarding cross-sectional detection [31] and prediction of AD [32].

Depressive symptoms were rated by the interviewer with the Montgomery Asberg Depression Rating Scale (MADRS) [33]. The MADRS consists of 10 items which are scored from 0 to 6 after a clinical interview. It is well established in psychogeriatric and AD studies [34]. A cut-off score of 13 points is suggested for mild depression. Instrumental activities of daily living were assessed with the Bayer-Activities of Daily Living Scale (BADL), a 25-item, informant-rated questionnaire developed to assess deficits in the performance of everyday activities in patients with MCI or mild-to-moderate dementia [35].

### Definition of MCI and incident AD

All diagnoses were established in a consensus conference between physicians and neuropsychologists at each site. The diagnosis of MCI was made according to the consensus criteria proposed in 2004 by the International Working Group on MCI [2]: (1) subjective and/or informant report about cognitive decline, (2) evidence of an impairment on objective cognitive test, (3) no or only minor impairments in instrumental activities of daily living (BADL score <4), and (4) not demented. Criterion (2) was met if patients showed a cognitive deficit of more than 1SD below age- and education-adjusted norms in at least one subtest of the CERAD-NP battery or in the Wechsler-Memory-Scales Logical Memory II subtest. The diagnosis of incident AD was made according to the NINCDS/ADRDA criteria for probable Alzheimer's disease [36].

### Classification of participants into “MCI with memory concerns” vs. “MCI without memory concerns”

Patients were classified as “MCI with memory concerns” (MC+) or “MCI without memory concerns” (MC-) according to their response to the following standardized question [6]: “Do you feel like your memory has become worse”. Possible answers were: (1) “No”, (2) “Sometimes, but this does not worry me”, (3) “Yes, that worries me”, (4) “Yes, that worries me seriously”. Answers (1) and (2) were combined to the MC- and answers (3) and (4) to the MC+ group, respectively.

The question and response categories were read aloud to patients by the interviewer as part of the initial assessment prior to neuropsychological testing. Duration of MC was not assessed in this study.

The standardized question on memory concerns was not used for the initial diagnosis of MCI but only for division into groups of MC+ and MC- patients respectively. The criterion of subjective report on cognitive decline required for the diagnosis of MCI could be provided either by the subject and/or by an informant according to the criteria of the International Working Group on MCI [2]. Thus the MC+ group constitutes a subgroup of MCI patients who themselves, when questioned in person with a standardized item, report memory decline which they appraise as particularly worrying. MC as operationalized here thus extend beyond the subjective report about cognitive decline as part of the general MCI criteria. Patients in the second response category “sometimes, but this does not worry me” were therefore assigned to the MC- group. We also refrained from keeping the four categories separate as this would have prevented the detailed analysis and straightforward interpretation of moderating effects between categorical (MC+ vs. MC-) and continuous (memory performance) variables, also due to limited number of participants answering “No” to the question on experienced memory decline. However, we report descriptive statistics of interest (conversion rates and memory performance) for all subgroups.

### Statistical analysis

Differences between groups were evaluated using independent sample t-tests for continuous and Chi^2^-test for categorical variables, respectively. Risk of incident AD was evaluated using stepwise Cox Proportional-Hazards regression analyses (SPSS-Version-20). Hazard Ratios (HR) with corresponding 95% Confidence Intervals (CI) are reported. Continuous predictors were age, years of education and the CERAD-NP delayed recall score (CERAD-DR). These were mean-centred prior to analysis by subtracting the respective sample mean from each observed value. Categorical predictors were gender, ApoE4-status (no E4 allele vs. presence of one or two E4 alleles) and group-status (MC- vs. MC+ group). In step 1 we entered age, gender, education, ApoE4 plus the CERAD-DR in the model. In step 2 we added group-status as an additional variable, to test the hypothesis that MC contribute to the risk of incident AD over time after controlling for objective memory impairment. In step 3 we added the linear interaction term of group-status and memory performance (group-status*CERAD-DR) to the model to test the hypothesis that the impact of MC on risk of future AD is moderated by the level of objective memory impairment. In an additional analysis we added the MADRS score in step 1 to control for depressive symptoms as a possible confounder.

Eight hundred and thirteen MCI patients were included at baseline in the longitudinal observational study. For the present analyses we included patients with a MMSE score between 24 and 30 (inclusive) and excluded patients with incomplete clinical or neuropsychological data required for the classification of subgroups and for statistical analysis. We further excluded those without information on ApoE4 genotype and those who withdrew early from the study without at least one follow-up visit at 12 months after baseline. Application of these criteria resulted in a sample of 454 MCI patients eligible for the present analyses. Thirty-seven patients (8.1%) converted to dementia other than AD during follow-up. We excluded these cases for the present analysis as our focus was on the impact of MC on incident AD. The final sample had a size of n = 417 MCI patients. Dropout analysis revealed that the group of patients excluded due to missing baseline data or lack of follow-up were older on average (M_excluded_ = 68.8, SD = 8.73; M_included_ = 65.6, SD = 7.93; p<0.05) but had only slightly lower MMSE mean scores (M_excluded_ = 27.3, SD = 1.72; M_included_ = 27.7, SD = 1.66; p<0.05). The two groups did not differ regarding years of education, gender distribution and expression of memory concerns (i.e. distribution of MC+ vs. MC-).

## Results

### Descriptive statistics of the sample

Of the 417 included patients, 19 patients (4.6%) responded “No” to the question on experienced memory decline, 93 (22.3%) answered “Sometimes, but this does not worry me”, 211 (50.6%) answered “Yes, that worries me” and 94 (22.5%) answered “Yes, that worries me seriously”. Thus, 112 (26.9%) patients were classified as MC- and 305 (73.1%) as MC+. The two groups did not differ in demographical variables, frequency of ApoE4 status, MMSE score, memory- or overall cognitive impairment on the CERAD-NP and mean follow-up time. MC+ patients showed higher scores on the MADRS scale and slightly higher BADL scores (Table 1).

**Table 1:** Description of the sample.

	Total Sample (n = 417 MCI patients)	MC- group (n = 112 MCI patients)	MC+ group (n = 305 MCI patients)	MC- vs. MC+ group	
				**Cohen's d**	**p-value**
Age (M, SD)	65.6 (7.93)	66.3 (8.70)	65.4 (7.63)	0.11	0.341
Years of Education (M, SD)	12.6 (2.84)	12.8 (2.81)	12.5 (2.85)	0.12	0.270
MMSE-Score (M, SD)	27.6 (1.66)	27.6 (1.62)	27.7 (1.67)	−0.06	0.617
CERAD Delayed Recall (M, SD)	5.3 (2.21)	5.3 (2.15)	5.4 (2.23)	−0.03	0.766
CERAD Total Score (M, SD)	73.3 (10.8)	73.4 (10.9)	73.2 (10.7)	0.02	0.888
MADRS (M, SD)	7.93 (6.34)	5.13 (5.01)	8.95 (6.47)	−0.63	<0.001
BADL-Score (M, SD)	2.16 (1.29)	1.96 (1.37)	2.23 (1.26)	−0.21	0.061
Follow-Up time in months (M, SD)	27.6 (9.85)	28.5 (10.5)	27.3 (9.61)	0.12	0.304
Time to Conversion in months (M, SD)	19.1 (7.80)	20.8 (7.42)	18.8 (7.87)	0.27	0.422
				**Chi^2^**	**p-value**
Female gender (n, %)	170 (40.8)	42 (37.5)	128 (42.0)	0.68	0.411
Positive ApoE4-status (n, %)	158 (37.9)	44 (39.3)	114 (37.4)	0.13	0.722
Conversion to AD (n, %)	74 (17.7)	11 (9.8)	63 (20.7)	6.59	0.01

*Note*. P-values are derived from independent sample t-tests (2-sided) for comparison of continuous variables, and from Chi^2^-tests for categorical variables. AD  =  Alzheimer's Dementia, BADL  =  Bayer-Activities of Daily Living Scale, CERAD  =  Consortium to Establish a Registry for Alzheimer's Disease, M  =  Mean, MADRS  =  Montgomery Asberg Depression Rating Scale, MMSE  =  Mini-Mental-State-Examination, MCI  =  Mild Cognitive Impairment, MC-  =  MCI patients without Memory Concerns, MC+  =  MCI patients with Memory Concerns, SD  =  Standard deviation.

### Risk of AD

Seventy-four patients (17.7%) developed incident AD within a mean follow-up time of 27.6 months. The incidence rate differed significantly between groups (9.8% vs. 20.7% for the MC- and MC+ group respectively). Incidence rates according to the individual response categories of the question on experienced memory decline were 6 out of 19 (31.6%) in the “No” category, 5 out of 93 (5.4%) in the category “Sometimes, but this does not worry me”, 42 out of 211 (19.9%) in the category “Yes, that worries me”, and 21 out of 94 (22.3%) in the category “Yes, that worries me seriously”. With regard to memory performance, the patients answering “No” had the lowest mean CERAD-DR scores (M = 4.37, SD = 2.63) while patients in the other categories displayed better and similar mean CERAD-DR scores (category “Sometimes, but this does not worry me”: M = 5.48, SD = 2.01; category “Yes, that worries me”: M = 5.29, SD = 2.16; category “Yes, that worries me seriously”: M = 5.53, SD = 2.21). Mean CERAD-DR performance in the group of patients answering “No” was significantly lower compared to that of patients in the other three response categories (t = 1.99, df = 415, p = 0.048).

Results of the Cox Proportional-Hazards regression models are presented in Table 2. In step 1, positive ApoE4 status (HR = 1.89, 95% CI: 1.18–3.02) and lower CERAD-DR performance (HR = 0.63, 95% CI: 0.56–0.71) were associated with higher risk of developing incident AD, yielding acceptable model fit (Nagelkerkes R^2^ = 0.262).

**Table 2:** Risk of incident Alzheimer's Dementia: Results from hierarchically formulated multivariate Cox proportional hazard regression models.

		Model Statistics				Predictor Statistics						
		M2LL Chi^2^	Δ-Chi^2^ (df)	p-value	Nagelkerke R2 (%)	B	SE	Wald-Chi^2^	p-value	HR	95% CI for HR	
											Lower	Upper
**Step 1**: Model with	**Model variables**	723.5	107.2 (5)	0.000	26.2							
covariates and	Age					0.03	0.02	2.33	0.127	1.03	0.99	1.06
CERAD-DR as	Female gender					0.26	0.24	1.17	0.279	1.29	0.81	2.05
predictors	Education					−0.05	0.04	1.52	0.218	0.95	0.88	1.03
	Positive ApoE4 status					0.63	0.24	6.95	0.008	1.89	1.18	3.02
	CERAD-DR					−0.47	0.06	56.11	0.000	0.63	0.56	0.71
**Step 2**: MC added as	**Model variables**	713.9	9.5 (1)	0.002	28.3							
predictor	Age					0.03	0.02	3.34	0.068	1.03	1.00	1.07
	Female gender					0.11	0.24	0.22	0.641	1.12	0.70	1.80
	Education					−0.05	0.04	1.77	0.183	0.95	0.88	1.03
	Positive ApoE4 status					0.61	0.24	6.39	0.011	1.85	1.15	2.97
	CERAD-DR					−0.46	0.06	55.87	0.000	0.63	0.56	0.71
	Presence of MC					0.93	0.33	7.87	0.005	2.55	1.33	4.89
**Step 3**: added	**Model variables**	709.1	4.8 (1)	0.028	29.3							
Interaction between	Age					0.03	0.02	2.97	0.085	1.03	1.00	1.07
CERAD-DR and	Female gender					0.16	0.24	0.44	0.506	1.18	0.73	1.90
MC	Education					−0.05	0.04	1.31	0.252	0.95	0.88	1.03
	Positive ApoE4 status					0.64	0.24	6.95	0.008	1.89	1.18	3.05
	CERAD-DR					−0.83	0.20	17.36	0.000	0.44	0.30	0.65
	Presence of MC					1.95	0.70	7.73	0.005	7.01	1.78	27.67
	**Linear Interaction**: CERAD-DR * MC					0.41	0.21	4.00	0.046	1.51	1.01	2.25

*Note*. M2LL of the Intercept model  = 830.6. Details of the modeling process are given in the methods section. The HR for the CERAD-DR is below one as it represents the HR for a one unit increase in CERAD-DR scores (i.e. for better memory performance). Lower CERAD-DR scores are therefore associated with a higher risk of developing incident AD. B  =  Beta-Coefficient of the predictor, CI  =  Confidence Interval, CERAD-DR  =  Delayed Recall of the Consortium to Establish a Registry for Alzheimer's Disease Neuropsychological Assessment Battery, HR  =  Hazard Ratio, M2LL  =  Minus-Two-Log-Likelihood, MC  =  Memory Concerns, SE  =  Standard Error for B.

Group-status (MC- vs. MC+) was entered in step 2 of the analysis. In addition to CERAD-DR and ApoE4, presence of MC (i.e. belonging to the MC+ group) was also associated with an increased risk of future AD (HR = 2.55, 95% CI: 1.33–4.89) and significantly increased model fit (Δ-Chi^2^ = 9.5, df = 1, p = 0.002, change in Nagelkerke's R^2^ = 2.1%). Thus, the hypothesis that presence of MC does individually contribute to the risk of future AD after controlling for objective memory impairment, was supported by the results of the regression analyses.

**Figure 1:**
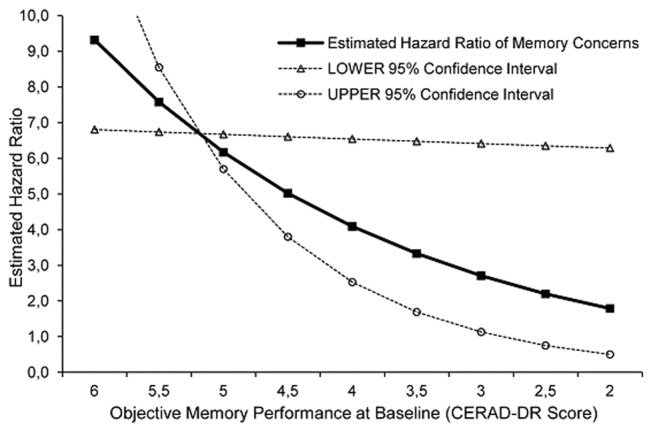
The impact of memory concerns on the risk of future Alzheimer's Dementia is moderated by objective memory performance at baseline. *Note*. The impact of memory concerns on the risk of future Alzheimer's Dementia, expressed in terms of the Hazard Ratio (HR) for the predictor “memory concerns”, is plotted as a function of objective memory performance at baseline, i.e. the interaction effect between memory concerns and objective memory performance is depicted. Values are derived from the multivariate Cox-proportional Hazard Regression analysis (see Table 2, model step 3: HR of the interaction-term  = 1.51, 95% Confidence Interval: 1.01–2.25). The black solid line corresponds to the estimated HR-value  = 1.51 of the interaction effect. The two dotted lines represent the functional curves that result when the boundary HR-values of the lower 95% Confidence Interval ( = 1.01) or upper 95% Confidence Interval ( = 2.25) respectively, are inserted as numbers to plot the interaction effect. CERAD-DR  =  Delayed Recall of the Consortium to Establish a Registry for Alzheimer's Disease Neuropsychological Assessment Battery.

The third step of the regression model included the interaction term of group-status and CERAD-DR. The overall model fit was again improved by inclusion of the interaction term (Δ-Chi^2^ = 4.8, df = 1, p = 0.028, change in Nagelkerke's R^2^ = 1%), supporting the hypothesis that the impact of MC on risk of future AD varies with the severity of objective memory impairment. The HR-value of the interaction term is greater than one (HR = 1.51, 95% CI: 1.01–2.25), which means that the impact of MC on the risk of future AD increases with higher memory performance and decreases with lower memory performance with an estimated factor of 1.5 per word. This moderating effect is depicted in Figure 1 (black solid line) where on the Y axis the estimated HR of MC is plotted as a function of memory performance (CERAD-DR). As can be seen here, the HR of MC decreases with decreasing memory performance, i.e. when moving from left to right along the X axis.

The additional analysis with the MADRS score as a predictor added in step 1 of the modelling process revealed that depressive symptoms were not associated with risk of future AD (p = 0.56) and did not alter the results reported above.

## Discussion

In the present study we found that MC, which extend beyond the subjectively experienced memory decline that is part of the general MCI criteria set, were associated with an increased risk of incident AD. This main effect of MC is of importance as it suggests that reported concerns regarding self-perceived memory decline (rather than just self-report without associated concerns) are predictive for future AD in the MCI stage. We suggest that the magnitude of this main effect (about two-fold increased risk in the MC+ group) is of clinical relevance. Our findings are in line with results from an independent population-based study which found that self-perceived memory decline with reported concerns is associated with a higher risk of incident AD than the mere notion of worsening memory (without concerns) [19], [20]. These results also suggest that AD related memory decline might be experienced in a different quality (i.e. as more serious and therefore worrying) compared to memory decline related to other factors such as normal aging. As an alternative hypothesis, proneness to psychological distress, a trait which has been reported as a risk factor for AD [37], might also be associated with a higher proneness to worry about self-perceived memory decline. If true, this could also explain the higher risk of incident AD associated with endorsing worries about worsening memory. We also stress that the main effect of MC does not imply that MCI patients without concerns about worsening memory are of no risk of future AD, but our data suggest that their risk is lower at a group level. Interestingly, in the small patient group who answered “No” to the question on experienced memory decline the conversion rate was highest and the memory performance level was lowest.

We also observed an interaction effect between MC and objective memory performance. The impact of MC on risk of future AD was highest for patients with very mild memory impairment and decreased with increasing memory impairment. Compared to the main effect of MC, this interaction effect was less strong. While this impedes a direct clinical applicability (e.g. for prediction in the individual case), it still highlights that at a group level MC and objective memory impairment interact in the course of AD. We suggest that this interaction between MC and memory performance is meaningful in several ways. Firstly, at the stage of very mild memory impairment, the assessment of self-perceived and worrying intra-individual decline might further contribute to AD prediction in addition to cross-sectional impairment on tests. This is of relevance as it highlights the particular value of self-reported memory decline with associated worries at the stage of very mild impairment [20].

Secondly, the effect of decreasing predictive validity of MC with increasing memory impairment may be caused by the reduction of self-perceived insight into symptoms at later stages of MCI. In this regard, we observed the highest conversion rate (31.6%) in the group answering “No” to the MC question, i.e. in those patients who were neither concerned about worsening memory nor reported any experienced memory decline at all. These patients also had the lowest CERAD-DR performance in the studied sample which is consistent with this potential explanation. Our observation is in line with results from a recent brain ^18^F-FDG-PET imaging study in a sample of single- and multidomain amnestic MCI patients (memory performance of <1.5 SD below norm), which also included an assessment of awareness [27]. Patients with poor awareness of their memory deficits showed a hypometabolic pattern similar to that of patients with early AD, suggesting that unawareness of memory deficits in MCI is linked to a more progressed pathology. Vogel et al. [24] studied a group of amnestic MCI patients with more severe memory impairment (<2SD below norm). They found similar levels of reduced awareness for this MCI group compared to a group of AD patients and observed lower MMSE scores to be associated with lower levels of awareness. Furthermore, one recent study has shown that, in the group of MCI patients, subjective cognitive complaints decreased with increasing cognitive impairment [18]. Based on these empirical data, we propose that anosognosia, which is a well-known clinical sign of AD, might occur at the stage of late MCI. At the stage of very mild MCI, before this loss of valid self-perception, the presence of MC is predictive of future AD. This is in agreement with several studies showing that subjective memory decline in individuals with normal cognitive function is also predictive for AD [6]–[8]; [19], [20].

Depressive symptoms did not predict risk of future AD in the present study and inclusion of depressive symptoms as a possible confounding variable did not alter the effects for objective memory impairment and MC. It is important to note, that although the MC+ group scored higher on the MADRS, their mean MADRS score reflected only very mild depressive symptoms and did not correspond to the clinical diagnosis of a major depression. ApoE4 status was associated with a higher risk of incident AD which is in line with recent studies [38], [39]. However, frequencies of ApoE4 did not differ between the MC+ and MC- group. Results remained similar when ApoE4 was not accounted for in the models and we did not observe an interaction between MC and ApoE4 with regard to risk of incident AD in additional post-hoc analyses (data not shown). ApoE4 and MC thus independently contributed to risk of AD in the present sample. We also controlled for level of education in our analysis. Regarding the interplay of education and memory concerns, results from a large population based cohort study of non-demented elderly suggest that the clinical relevance of subjective memory complaints might be higher in individuals with higher educational background [40]. We also tested for an interaction between memory concerns and level of education in our analysis but did not find such an effect (data not shown). Differences in samples and design (i.e. community based cohort of non-demented elderly vs. memory clinic MCI sample in our study) might have contributed to these discrepant findings.

Our results are different to those of other studies which did not find a clear association between self-reports of memory decline and incident AD [4], [5]. However, besides differences regarding samples and assessment of self-reported memory decline, these studies did also include informant reports in their predictive models. Therefore the comparability of our results to these studies is limited and we acknowledge the lack of informant reports in our study as a limitation.

A strength of the present study is the large number of neuropsychologically well characterized patients who met criteria for MCI [2]. Within these criteria we set the cutoff for cognitive impairment at 1SD below the normative mean. This procedure is in line with recently established study protocols of large studies, e.g. ADNI-2 where recruitment was extended to early (amnestic) MCI patients with very mild memory impairment (<1.0 SD below the norm) [41]. The present sample therefore enabled us to test the specific contribution of MC for risk of AD at different stages of memory impairment within the MCI spectrum.

This study has limitations. The present sample reflects MCI patients with at least very mild impairment in one cognitive domain. Therefore the present results concerning the prognostic value of MC at different levels of memory impairment only refer to the MCI spectrum and not to cognitively unimpaired individuals. Secondly, we focused on memory concerns only (rather than concerns about other cognitive domains or cognition in general) and on AD as the outcome. It is important to note that other cognitive domains beyond memory can also be affected in MCI due to Alzheimer's disease [3]. Thirdly, data on duration of MC and on discrepancies between the informant and the patient regarding the report of MC was not available to us. Finally, our sample reflects a memory clinic population and the transfer to population-based cohort or volunteer samples may not be valid. Dropout analysis also revealed that the patients included in this study were three years younger on average compared to those excluded due to baseline missing data or lack of follow-up. However the two groups differed only slightly regarding baseline cognitive functioning and, more importantly, the groups did not differ in the expression of MC (73.1% MC+ in the study sample vs. 74.8% MC+ in those excluded from the analysis; p = 0.661). Thus, although a small selection bias was observed in our data, we consider the main results of our study not confounded by this bias.

In conclusion, the present study highlights a dynamic of the impact of MC as a predictor for incident AD in MCI patients. The results may have implications for clinicians working with elderly patients at risk of AD, but also for the design of early intervention trials in Alzheimer's disease. MC should be taken seriously as a risk indicator for future AD, especially in cases where neuropsychological test results are at the border between normal and impaired.

## References

[1] Petersen RC (2004) Mild cognitive impairment as a diagnostic entity. J Intern Med 256 (3): 183–194. 15324362 10.1111/j.1365-2796.2004.01388.x

[2] Winblad B, Palmer K, Kivipelto M, Jelic V, Fratiglioni L, et al (2004) Mild cognitive impairment – beyond controversies, towards a consensus: report of the International Working Group on Mild Cognitive Impairment. J Intern Med 256 (3): 240–246. 15324367 10.1111/j.1365-2796.2004.01380.x

[3] Albert MS, DeKosky ST, Dickson D, Dubois B, Feldman HH, et al (2011) The diagnosis of mild cognitive impairment due to Alzheimer's disease: Recommendations from the National Institute on Aging-Alzheimer's Association workgroups on diagnostic guidelines for Alzheimer's disease. Alzheimers Dement 7 (3): 270–279. 21514249 10.1016/j.jalz.2011.03.008 PMC3312027

[4] Tierney MC, Szalai JP, Snow WG, Fisher RH (1996) The prediction of Alzheimer disease. The role of patient and informant perceptions of cognitive deficits. Arch. Neurol 53 (5): 423–427. 8624217 10.1001/archneur.1996.00550050053023

[5] Rabin LA, Wang C, Katz MJ, Derby CA, Buschke H, et al (2012) Predicting Alzheimer's disease: neuropsychological tests, self-reports, and informant reports of cognitive difficulties. J Am Geriatr Soc 60 (6): 1128–1134. 22690986 10.1111/j.1532-5415.2012.03956.x PMC3375855

[6] Geerlings MI, Jonker C, Bouter LM, Adèr HJ, Schmand B (1999) Association between memory complaints and incident Alzheimer's disease in elderly people with normal baseline cognition. Am J Psychiatry 156 (4): 531–537. 10200730 10.1176/ajp.156.4.531

[7] Reisberg B, Shulman MB, Torossian C, Leng L, Zhu W (2010) Outcome over seven years of healthy adults with and without subjective cognitive impairment. Alzheimers Dement 6 (1): 11–24. 20129317 10.1016/j.jalz.2009.10.002 PMC3873197

[8] Jessen F, Wiese B, Bickel H, Eiffländer-Gorfer S, Fuchs A, et al (2011) Prediction of dementia in primary care patients. PLoS ONE 6 (2): e16852. 21364746 10.1371/journal.pone.0016852 PMC3041758

[9] Saykin AJ, Wishart HA, Rabin LA, Santulli RB, Flashman LA, et al (2006) Older adults with cognitive complaints show brain atrophy similar to that of amnestic MCI. Neurology 67 (5): 834–842. 16966547 10.1212/01.wnl.0000234032.77541.a2 PMC3488276

[10] Mosconi L, Santi Sde, Brys M, Tsui WH, Pirraglia E, et al (2008) Hypometabolism and altered cerebrospinal fluid markers in normal apolipoprotein E E4 carriers with subjective memory complaints. Biol. Psychiatry 63 (6): 609–618. 10.1016/j.biopsych.2007.05.030 PMC2386268 17720148

[11] Visser PJ, Verhey F, Knol DL, Scheltens P, Wahlund L, et al (2009) Prevalence and prognostic value of CSF markers of Alzheimer's disease pathology in patients with subjective cognitive impairment or mild cognitive impairment in the DESCRIPA study: a prospective cohort study. Lancet Neurol 8 (7): 619–627. 19523877 10.1016/S1474-4422(09)70139-5

[12] Chételat G, Villemagne VL, Bourgeat P, Pike KE, Jones G, et al (2010) Relationship between atrophy and beta-amyloid deposition in Alzheimer disease. Ann. Neurol 67 (3): 317–324. 20373343 10.1002/ana.21955

[13] Scheef L, Spottke A, Daerr M, Joe A, Striepens N, et al (2012) Glucose metabolism, gray matter structure, and memory decline in subjective memory impairment. Neurology 79 (13): 1332–1339. 22914828 10.1212/WNL.0b013e31826c1a8d

[14] Amariglio RE, Becker JA, Carmasin J, Wadsworth LP, Lorius N, et al (2012) Subjective cognitive complaints and amyloid burden in cognitively normal older individuals. Neuropsychologia 50 (12): 2880–2886. 22940426 10.1016/j.neuropsychologia.2012.08.011 PMC3473106

[15] Mielke MM, Wiste HJ, Weigand SD, Knopman DS, Lowe VJ, et al (2012) Indicators of amyloid burden in a population-based study of cognitively normal elderly. Neurology 79 (15): 1570–1577. 22972644 10.1212/WNL.0b013e31826e2696 PMC3475629

[16] Reid LM, Maclullich AMJ (2006): Subjective memory complaints and cognitive impairment in older people. Dement Geriatr Cogn Disord 22 (5–6): , 471–485. 10.1159/000096295 17047326

[17] Buckley R, Saling MM, Ames D, Rowe CC, Lautenschlager NT, Macaulay SL, et al (2013) Factors affecting subjective memory complaints in the AIBL aging study: biomarkers, memory, affect, and age. Int. Psychogeriatr 25 (08): 1307–1315. 23693133 10.1017/S1041610213000665

[18] Grambaite R, Hessen E, Auning E, Aarsland D, Selnes P, et al (2013) Correlates of Subjective and Mild Cognitive Impairment: Depressive Symptoms and CSF Biomarkers. Dement Geriatr Cogn Dis Extra 3 (1): 291–300. 24174924 10.1159/000354188 PMC3808228

[19] Jessen F, Wiese B, Bachmann C, Eifflaender-Gorfer S, Haller F, et al (2010) Prediction of Dementia by Subjective Memory Impairment: Effects of Severity and Temporal Association With Cognitive Impairment. Arch Gen Psychiatry 67 (4): 414–422. 20368517 10.1001/archgenpsychiatry.2010.30

[20] Jessen F, Wolfsgruber S, Wiese B, Bickel H, Mösch E, Kaduszkiewicz H, et al. (2014): AD dementia risk in late MCI, in early MCI, and in subjective memory impairment. Alzheimers Dement 10 (1): , 76–83. 10.1016/j.jalz.2012.09.017 23375567

[21] Mitchell AJ (2008) The clinical significance of subjective memory complaints in the diagnosis of mild cognitive impairment and dementia: a meta-analysis. Int. J. Geriat. Psychiatry 23 (11): 1191–1202. 10.1002/gps.2053 18500688

[22] Roberts J, Clare L, Woods R (2009) Subjective Memory Complaints and Awareness of Memory Functioning in Mild Cognitive Impairment: A Systematic Review. Dement Geriatr Cogn Disord 28 (2): 95–109. 19684399 10.1159/000234911

[23] Vogel A, Stokholm J, Gade A, Andersen BB, Hejl A, et al (2004) Awareness of Deficits in Mild Cognitive Impairment and Alzheimer's Disease: Do MCI Patients Have Impaired Insight. Dement Geriatr Cogn Disord 17 (3): 181–187. 14739542 10.1159/000076354

[24] Vogel A, Hasselbalch SG, Gade A, Ziebell M, Waldemar G (2005) Cognitive and functional neuroimaging correlate for anosognosia in Mild Cognitive Impairment and Alzheimer's disease. Int. J. Geriat. Psychiatry 20 (3): 238–246. 10.1002/gps.1272 15717342

[25] Galeone F, Pappalardo S (2011) Chieffi S, Iavarone A, Carlomagno S (2011) Anosognosia for memory deficit in amnestic mild cognitive impairment and Alzheimer's disease. Int. J. Geriat. Psychiatry 26 (7): 695–701. 10.1002/gps.2583 21495076

[26] Kalbe E, Salmon E, Perani D, Holthoff V, Sorbi S, et al (2005) Anosognosia in very mild Alzheimer's disease but not in mild cognitive impairment. Dement Geriatr Cogn Disord 19 (5–6): 349–356. 15802909 10.1159/000084704

[27] Nobili F, Mazzei D, Dessi B, Morbelli S, Brugnolo A, et al (2010) Unawareness of Memory Deficit in Amnestic MCI: FDG-PET Findings. J Alzheimers Dis 22 (3): 993–1003. 20858977 10.3233/JAD-2010-100423

[28] Kornhuber J, Schmidtke K, Fr&ouml, lich L, Perneczky R, et al (2009) Early and Differential Diagnosis of Dementia and Mild Cognitive Impairment. Dement Geriatr Cogn Disord 27 (5): 404–417. 19339779 10.1159/000210388

[29] Morris JC, Heyman A, Mohs RC, Hughes JP, van Belle G, et al (1989) The Consortium to Establish a Registry for Alzheimer's Disease (CERAD). Part I. Clinical and neuropsychological assessment of Alzheimer's disease. Neurology 39 (9): 1159–1165. 2771064 10.1212/wnl.39.9.1159

[30] Folstein MF, Folstein SE, McHugh PR (1975) “Mini-mental state”: a practical method for grading the cognitive state of patients for the clinician. J Psychiatr Res 12 (3): 189–198. 1202204 10.1016/0022-3956(75)90026-6

[31] Sotaniemi M, Pulliainen V, Hokkanen L, Pirttilä T, Hallikainen I, et al (2012) CERAD-neuropsychological battery in screening mild Alzheimer's disease. Acta Neurol Scand 125 (1): 16–23. 21198445 10.1111/j.1600-0404.2010.01459.x

[32] Wolfsgruber S, Jessen F, Wiese B, Stein J, Bickel H, et al.. (2013) The CERAD Neuropsychological Assessment Battery Total Score Detects and Predicts Alzheimer Disease Dementia with High Diagnostic Accuracy. Am J Geriatr Psychiatry. 10.1016/j.jagp.2012.08.021 23759289

[33] Montgomery SA, Asberg M (1979) A new depression scale designed to be sensitive to change. Br J Psychiatry 134: 382–389. 444788 10.1192/bjp.134.4.382

[34] Müller-Thomsen T, Arlt S, Mann U, Maβ R, Ganzer S (2005) Detecting depression in Alzheimer's disease: evaluation of four different scales. Arch Clin Neuropsychol 20 (2): 271–276. 15708735 10.1016/j.acn.2004.03.010

[35] Hindmarch I, Lehfeld H, Jongh P de, Erzigkeit H (1998) The Bayer Activities of Daily Living Scale (B-ADL). Dement Geriatr Cogn Disord 9 (Suppl. 2)20–26. 10.1159/000051195 9718231

[36] McKhann G, Drachman D, Folstein M, Katzman R, Price D, et al (1984) Clinical diagnosis of Alzheimer's disease: Report of the NINCDS-ADRDA Work Group* under the auspices of Department of Health and Human Services Task Force on Alzheimer's Disease. Neurology 34 (7): 939. 6610841 10.1212/wnl.34.7.939

[37] Wilson RS, Evans DA, Bienias JL, Mendes de Leon CF, Schneider JA, Bennett DA (2003): Proneness to psychological distress is associated with risk of Alzheimer's disease. Neurology 61 (11): , 1479–1485. 10.1212/01.wnl.0000096167.56734.59 14663028

[38] Xu W, Caracciolo B, Wang H, Santoni G, Winblad B, et al (2013) Accelerated Progression from Mild Cognitive Impairment to Dementia Among APOE ε4ε4 Carriers. J Alzheimers Dis 33 (2): 507–515. 23247007 10.3233/JAD-2012-121369

[39] Espinosa A, Alegret M, Valero S, Vinyes-Junqué G, Hernández I, et al (2013) A Longitudinal Follow-Up of 550 Mild Cognitive Impairment Patients: Evidence for Large Conversion to Dementia Rates and Detection of Major Risk Factors Involved. J Alzheimers Dis 34 (3): 769–780. 23271318 10.3233/JAD-122002

[40] van Oijen M, Jong FJ de, Hofman A, Koudstaal PJ, Breteler MMB (2007) Subjective memory complaints, education, and risk of Alzheimer's disease. Alzheimers Dement 3 (2): 92–97. 19595922 10.1016/j.jalz.2007.01.011

[41] Aisen PS, Petersen RC, Donohue MC, Gamst A, Raman R, et al (2010) Clinical core of the Alzheimer's disease neuroimaging initiative: Progress and plans. Alzheimers Dement 6 (3): 239–246. 20451872 10.1016/j.jalz.2010.03.006 PMC2867843

